# Exploring the online learning experience of first-year speech–language pathology students in a Johannesburg-based university

**DOI:** 10.4102/sajcd.v69i2.914

**Published:** 2022-07-29

**Authors:** Nancy Barber, Jenna Sher

**Affiliations:** 1 Department of Speech-Language Pathology, Faculty of Humanities, University of the Witwatersrand, Johannesburg, South Africa

**Keywords:** COVID-19, online learning, pedagogy, first-year, resilience, educator, support, Johannesburg

## Abstract

**Background:**

Understanding the learning experiences of first-year speech–language pathology (SLP) students during the coronavirus disease 2019 (COVID-19) pandemic is essential to ensure that academic staff are able to support and enhance the transition from secondary to tertiary education. An understanding of the student experience could lead to improved support strategies that could be beneficial for the blended learning environment that the University of the Witwatersrand will be entering from 2022.

**Objectives:**

This research explored the experiences of first-year SLP students in online learning during the COVID-19 pandemic.

**Method:**

An exploratory mixed-method concurrent triangulation design was employed. Quantitative data were collected from likert scales. Qualitative data were collected from critical incident timelines. Themes were identified from both the Likert scales as well as the critical incident timelines using bottom-up thematic analysis.

**Results:**

The majority of participants reflected that their online learning through the pandemic in 2021 was successful. The themes that emerged from this year pertain to 2021 and the specific participants however, it provides an important insight that the students’ needs change during a year. As a lecturer, one needs to consider these evolving needs to ensure students have the support that they require to be successful in their learning.

**Conclusion:**

This research provided insights into the evolving nature of the support first-year SLP students require in the online learning space during the COVID-19 pandemic.

## Background

It has been established in research that the adjustment from secondary school to tertiary education can be challenging for students (Lekena & Bayaga, 2018). It is reported that dropout rates in the first year of university are high worldwide (Van Rooij, Jansen, & Van de Grift, 2017). It has also been found that a smooth transition from secondary school to university can increase the chances of students’ success, specifically in achievement and persistence (Lowe & Cook 2003; Rienties, Beausaert, Grohnert, Niemantsverdriet, & Kommers, 2012). Lekena and Bayaga (2018) identified that one needs to understand the first-year students’ perspectives so that their needs can be met to ensure that their potential is not wasted (Lekena & Bayaga, 2018). Although it is acknowledged that the first year of university is an important transition period for students, there is a paucity of understanding as to how to enhance success and persistence in tertiary education (Lekena & Bayaga, 2018). There is also limited knowledge as to how best to promote a positive first-year experience in order to facilitate the transition to tertiary education.

There is a well-known history in South Africa of marginalised populations who have not been provided with equitable educational services. To facilitate transformation, the University of the Witwatersrand has emphasised the need to transform the student demographics in terms of gender, race, socio-economic status and prior education so that students from more varied demographics have access to tertiary education and are supported appropriately to be able to obtain their degrees (University of the Witwatersrand Senior Executive Team, 2015). This transformation has led to students requiring different types of support to succeed. The transformation in the student body at the University of the Witwatersrand, especially in the speech–language pathology (SLP) department, has led to the need to transform the pedagogies employed. There have been several papers written highlighting the need to transform and decolonise the SLP curriculum in the South African context, and thus this is required for the context of the University of the Witwatersrand SLP department (Adams, Mupawose, Kelly, & Moonsamy, 2021; Kathard & Pillay, 2013; Khoza-Shangase & Mophosho, 2018, 2021; Moonsamy, Mupawose, Seedat, Mophosho, & Pillay, 2017; Morreira, Luckett, Kumalo, & Ramgotra, 2020; Pillay & Kathard, 2015, 2018).

The COVID-19 pandemic has been termed a disruptor of traditional education (Adedoyin & Soykar, 2020; Thurston, 2021). Online learning with synchronous and asynchronous tasks has been employed at the University of the Witwatersrand for the year 2021. This online learning environment needs to be considered for the first-year student who traditionally may find the adjustment to tertiary education challenging. Thus, for academic staff, the need to support the transition to tertiary education in the current state of learning is essential. The COVID-19 pandemic as an educational disruptor seems to have advanced the change in pedagogy more swiftly than in previous years (Thurston, 2021). Within education, there are a variety of pedagogies employed. Traditional educational pedagogy refers to teacher-centred pedagogy (Khalaf & Zin, 2018; Patel et al., 2021). The teacher is seen as the source of knowledge and students are seen as passive vessels that absorb knowledge (Patel et al., 2021). Behaviourism underpins the traditional educational pedagogy and suggests that learning occurs because of the influence of the teacher on students (Khalaf & Zin, 2018). Traditional educational pedagogy that is influenced by behaviourism has been criticised by constructivist learning theories (Khalaf & Zin, 2018). Constructivist learning theory emphasises that students construct knowledge during the learning process (Khalaf & Zin, 2018). Cognitive and constructivist approaches to learning are therefore student-centred (Khalaf & Zin, 2018).

Given the current circumstances of remote online learning in which education is occurring, educators have been challenged to integrate constructivist learning theory within the online learning space and assist students as they transition from secondary school to tertiary education via online learning. Understanding the learning experiences of first-year SLP students during the COVID-19 pandemic is essential to ensure that academic staff can support and enhance the transition from secondary to tertiary education. An understanding of the student experience could lead to improved support strategies that could be beneficial for the hybrid learning environment that the University of Witwatersrand will be entering from 2022.

## Aim

To explore the experiences of first-year SLP students in online learning during the COVID-19 pandemic.

## Objectives

To explore the experiences of first-year SLP students in online learning during the COVID-19 pandemic using a Likert scale and focus group.

To explore the critical incidents that occurred throughout the first year that impacted the SLP students’ experience of online learning during the pandemic, by means of a timeline.

To gain an understanding of when first-year SLP students require the most support during their first year of study.

## Methodology

This study was an exploratory mixed methods concurrent triangulation design. Quantitative data were collected from Likert scales. Qualitative data were collected from the critical incidents recorded on the timelines. This research study employed purposive sampling of first-year SLP students from 2021 at the University of the Witwatersrand. Twelve of 36 first-year SLP students completed the Likert scale and critical incident timeline. It must be noted that because of the small sample size, this study may not be representative of the learning experiences of the full cohort of students.

At the end of the academic year, all the students in the sample completed the Likert scale and the timeline reflecting the critical incidents retrospectively for all four academic blocks. Students completed a Likert scale reflecting on their online learning experience in the first-year curriculum specifically in Speech–Language Pathology I (SPPA1004A) (see Appendix 1). A timeline using the critical incident technique (CIT) was then completed. Critical incident technique includes reflecting on critical incidents, which are events that were positive or negative that occurred and made an impact on the student (Cunningham, De Brún, & McAuliffe, 2020). Critical incident technique is known to be a good reflection tool that can provide in-depth information regarding a participant’s experience (Cunningham et al., 2020). Students were instructed to plot any incidents (academic or personal) that occurred in each block that they felt had a significant impact on themselves as students, and they had to reflect on the reason that each incident was important to them (Cunningham et al., 2020). A timeline format can support reflection as it provides a visual structure that allows a participant to reconstruct an experience or incident and thus can enable comparison and evaluation of experiences (Kristiansen, Storlien, Mora, Krogstie, & Divitini, 2012). The students were provided with the instructions and timeline template in Appendix 2 as a framework for their reflection.

The data from the timelines were analysed using a bottom-up approach by employing the six steps of reflexive thematic analysis (TA) as described by Braun and Clarke (2021), which include generating codes, grouping them together, identifying patterns in the data and thus inferring the themes that are reflected in the patterns (Braun & Clarke, 2021).

The trustworthiness of the research was established using the four pillars of Lincoln and Guba (1985). The trustworthiness may be impacted by students completing the Likert scale and critical incident timelines retrospectively at the end of the year because of recall bias. Credibility was promoted through methodological and data triangulation. Transferability was established through purposive sampling and thick descriptions (Stahl & King, 2020). The dependability of the research was established through a peer debriefer and reflexive auditing, by means of the two researchers analysing the data separately and then reviewing and confirming the themes identified. The researchers were cognisant to adopt an attitude that reflected their own backgrounds and positionality, remaining aware of how this could influence the research process. This was achieved through detailed note-taking and reflective discourse.

### Ethical considerations

Ethical clearance to complete the research was obtained from the University of the Witwatersrand HREC (nonmedical) committee with the protocol number H21/11/47.

## Results and discussion

The results from the study will be discussed with regard to the themes that emerged from the Likert scale, and then these will be integrated with the themes from the critical incidents reported in the timelines.

## Online learning journey

**FIGURE 1:**
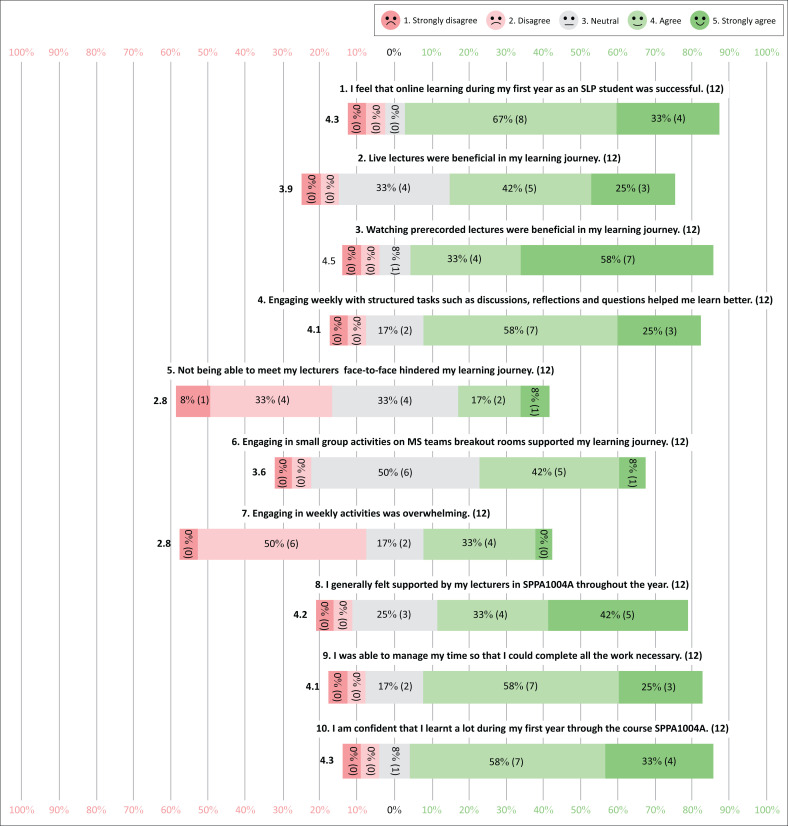
Visual representation of the Likert scale responses from the first-year speech–language pathology students.

Results from the Likert scale are illustrated in Figure 1 using descriptive statistics.

These results identified that students felt that their year of online learning was successful, and they felt confident that they had learnt a lot through the SPPA1004A course even though it was online, as reflected by responses in Statement 1 and Statement 10. The results also highlighted that students found prerecorded lectures beneficial in their learning journey (Statement 3), that weekly engagement tasks helped them to learn better (Statement 4) and that participating in weekly engagement tasks was not overwhelming (Statement 7). Live lectures and Microsoft Teams breakout rooms seemed to be beneficial but with minimally less impact than the aforementioned teaching and learning strategies, as can be observed in Statements 2 and 6. Positively, students also reflected that they felt generally supported by staff throughout the year (Statement 8) and that not meeting their lecturers face to face did not hinder their learning experience (Statement 5). In addition, students reflected that they had sufficient time management skills (Statement 9).

These results provide insight that students feel that they can learn successfully through online learning platforms when there are prerecorded lectures with engaging tasks to support their active learning. This insight is supported by adult learning principles, which validate that active learning pedagogies need to be carefully considered and employed to promote learning (Noffs & Wilson, 2021), and thus as educators we need to be mindful of designing and structuring our course to promote active learning.

## Critical incident themes

**FIGURE 2:**
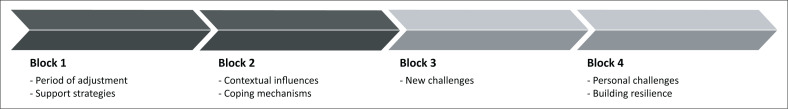
The themes per block for the academic year of 2021.

The thematic results from the timelines submitted were constructed based on the bottom-up thematic analysis of each academic block. At the University of the Witwatersrand, the academic year comprises four blocks of 7 weeks per block, Block 1 being the first block of the year and progressing to Block 4 at the end of the academic year. After Block 2 is the mid-year exam period, and after Block 4 is the final year exam period. During the analysis of critical incidents reported in the timelines, it was noted that there were specific themes that were inherent to each block, and the themes did not appear to be generalised across the entire year. Thus, when reporting these themes, they will be reported per block. Reporting them per block also provides insight for educators as to the changing support required by students across the academic year, as well as the variety of support that is possibly required by first-year students engaging in online learning. Figure 2 reflects the changing themes across the blocks of the 2021 academic year.

### Themes for block 1

#### Period of adjustment and support strategies

Two main themes emerged from the first academic block of study. Students identified a period of adjustment and supportive strategies as the most significant incidents of that block. The theme ‘period of adjustment’ included the following statements: ‘I was overwhelmed by the new environment’ (Student 4), ‘first few weeks of block were very difficult’ (Student 11) and ‘I was not familiarised with the way things are done on the university level and also struggled a bit navigating on an online platform’ (Student 12). Interestingly, the same students subsequently included statements that contributed to the theme of ‘supportive strategies’. They reported, respectively, that ‘it took some time for me to adjust and understand what was required’, ‘I was able to adapt quickly’ and ‘lectures gave tips on how to tackle each course productively, prioritise and use time wisely’.

Whilst students initially struggled with the transition into tertiary education, they were able to overcome these obstacles by means of their own resilience and support from peers and university staff. This is consistent with results from the Likert scale where students reflected that they felt generally supported by staff through the year. Similarly, a study by Picton and Kahu (2021) reported that students who utilised academic support services found them helpful and that support services positively influenced their self-efficacy and ultimately improved their academic performance. Support was also reported to positively influence students’ emotions and increase their sense of belonging to the university community (Picton & Kahu, 2021).

### Block 2 themes

#### Contextual influences and coping mechanisms and strategies

The main themes that emerged from Block 2 of the academic year were ‘contextual influences’ and ‘coping mechanisms and strategies’. Students described novel incidents associated with events that were specific to that particular point in time, as demonstrated by the following responses: ‘my whole family contracted COVID during the holiday’ (Student 9); ‘Block 2 was when we would start writing exams … for the first time’ (Student 11);’ and ‘load shedding occurred when I was writing my online exam’ (Student 12).

The second block of the academic year coincided with the June/July examination period. During this period in South Africa, there was the advent of the third wave of COVID-19 infections, from 01 June 2021 to 23 June 2021, driven mainly by the dominant delta variant (Maslo et al., 2021). Furthermore, the implementation of load shedding during this time further complicated the students’ ability to engage in online learning. Similarly, a study conducted by Mavhandu-Mudzusi, Mudau, Shandu and Ndou (2021) reported that intermittent power supply as a result of load shedding led to an unstable Internet connection and failure to power up their devices, preventing them from engaging in online learning.

Whilst the students reported difficulties because of contextual influences, they were able to employ coping mechanisms and strategies to overcome these obstacles. Student 9 reported that ‘[contracting COVID] set me back a little bit, but luckily I have completed my hours now’, whilst Student 12 commented on the lecturer’s supportive response to load shedding: ‘I emailed the lecturer to tell them about my issue; they were so calm about it and reassured me that there is nothing to worry about’. Successful student strategies can improve well-being, counter negative emotions and help foster positive ones, all of which facilitate students engaging more deeply with their learning (Picton & Kahu, 2021).

### Block 3 theme

#### New challenges

The main theme reflected in Block 3 was termed ‘new challenges’. The challenges presented in Block 3 were not related to the challenges reflected in the period of adjustment in Block 1, nor were they related to the contextual influences that were identified in Block 2. The theme of new challenges incorporated the type of learning strategies employed (case studies and group work) as well as students being tired. Educators in SPPA1004A employ a variety of pedagogies to engage students in the coursework and to facilitate active learning of the curriculum. The use of case studies and group work are tools that facilitate deep learning, as evidenced by increased reasoning and academic results when these pedagogies are employed (Chang & Brickman, 2018; Puri, 2020).

The use of case studies facilitates student-centred learning that promotes a deeper understanding of real-life complexities for a particular case, therefore assisting with bridging the theory-practice gap (Puri, 2020). Speech–language pathology students must be able to apply their learned knowledge to make and justify clinical decisions in novel situations (Mok, Whitehill, & Dodd, 2014). Exposing students to case studies in the first year may be complex, as Student 4 reported: ‘[Block 3] seemed a bit more complicated with the case studies – but I nonetheless enjoyed it’. However, as Puri (2020) explains, this pedagogy allows the development of skills required for understanding the real-life complexities of the cases that students will one day assess and treat.

Group work is also a useful pedagogy to increase the depth of learning (Chang & Brickman, 2018). However, in the study by Chang and Brickman (2018), students reflected that group work could be difficult because of the unequal contributions by group members, even though they found the cognitive and social support beneficial. Peer reviews and group contracts did not seem to assist with equal group member contribution in the study by Chang and Brickman (2018). Similar challenges were faced by our participants, as stated by Student 2: ‘In Block 3 I did not quite enjoy our assignment task. … Group work became exceedingly challenging due to group members not cooperating’.

The challenges of online group work are important to consider when designing learning for students in online learning during the COVID-19 pandemic. One needs to ensure the support for case studies and group work are consolidated so that students can use these pedagogies as deep learning experiences.

Another aspect of the theme of new challenges that emerged was fatigue. Student 12 stated that they were ‘starting to get emotionally, mentally and physically tired, but I kept pushing and reminded myself that I can do this and giving up is not worth it’. Tertiary online learning requires that students are psychologically and physically prepared to receive large volumes of information and to be able to successfully participate in self-regulated learning (Tugtekin, 2022). The use of online learning during the COVID-19 pandemic has led to psychological and physical fatigue in students because of factors such as excessive technology use, unconscious use of technology and low technology literacy (Tugtekin, Barut Tugtekin, Kurt, & Demir, 2020). Educators need to be aware of the psychological and physical exertion that online learning places on students to best support them during times of fatigue.

### Block 4 themes

#### Personal challenges and building resilience

The main themes reflected in Block 4 were ‘personal challenges’ and ‘building resilience’. The theme of personal challenges was made evident through critical incidents shared. Student 5 shared: ‘I fell behind with many of my assignments and work because I had lost my cousin to suicide and I struggled to process it’. Student 10 shared: ‘My uncle passed away … This had a negative impact on my mood’. Student 11 reported that ‘Block 4 was an emotional rollercoaster for me, personally’. Student 12 also reflected that ‘final [end of] year anxiety was getting to me’.

These participants’ experiences are corroborated by research conducted by Cénat et al. (2020). This research identified that the COVID-19 pandemic had impacted students’ lives in a diverse number of areas but that the main consequence of these impacts was fear, worry, anxiety and exacerbated stress (Cénat et al., 2020). This same concept can be seen in the aforementioned quotes from the participants. The COVID-19 pandemic has impacted the participants’ lives in diverse ways that have led to negative emotions. Cénat et al. (2020) identified a humanistic approach to sharing COVID-19 traumas based on caring, empathy and sharing of experiences that led to the development of coping strategies and resilience.

This humanistic approach identified by Cénat et al. (2020) leads to the second main theme of ‘building resilience’ that was identified in Block 4. Resilience is generally defined as the effective adaptation to stress, change or adversity (Flinchbaugh, Luth, & Li, 2015). Delany et al. (2015) described resilience as occurring when an individual is positively transformed by the adversity that they have experienced. During the COVID-19 pandemic, there has been considerable stress, change and adversity for first-year SLP students, as reflected in the personal challenges theme and the themes of the other blocks. However, participants reflected that they were able to overcome the challenges of online learning during the COVID-19 pandemic and be positively transformed by the challenges that they experienced. Student 11 stated:

‘[*L*]ooking back just makes me really emotional, but I managed to get through the block – even obtained distinctions. I believe what happened to me in Block 1, realising my potential as a student, played a huge role in how I dealt with Block 4.’

Student 9 reflected: ‘I was lucky enough to reach some of my academic goals and my hard work throughout the year paid off’.

Participants reflected on the importance of a caring, empathetic, humanistic approach to their teaching and learning experience. Student 12 stated that ‘there were support groups and lecturers … [and] also a support system to help us with any topics of the course that we were struggling with’. Student 3 reported that ‘lecturers were also incredibly supportive and showed recognition and attention to the concerns of students’. A supportive environment assisted with the development of the participants’ resilience. The impact of a supportive environment corroborates the findings of a scoping review of resilience in higher education students by Brewer et al. (2019). The scoping review revealed the need for educators to be aware of the organisational context, as this impacts the development of resilience in students (Brewer et al., 2019).

## Critical incident trends across the year

It was observed that the critical incidents reported over the blocks seemed to decrease across the year. Table 1 reflects the number of incidents reported per block that impact the students’ teaching and learning. The number of students reporting that a critical incident did not occur increased from Block 1 to Block 3, indicating that the students had fewer incidents that affected their learning as the year progressed. However, in Block 4, the students had more critical incidents occurring. This trend highlights that students appear to require more support at the beginning and at the end of an academic year. With the transition from high school to university, the beginning of the first-year experience is dotted with unexpected challenges and the transition towards becoming more mature (Lee, Ang, & Dipology-Ubanan, 2019). Towards the end of the first-year experience, students have a developed a new level of awareness and maturity that allows them to identify their difficulties with more ease and, at the same time, develop an expectation for a better learning environment (Lee et al., 2019). This is important for educators facilitating teaching and learning at those stages of the academic year to be mindful of.

**TABLE 1:** Number of students reporting critical incidents per block.

Block	Number of students reflecting that a critical incident did not occur	Number of students reflecting that a critical incident did occur
1	3	9
2	4	8
3	6	6
4	3	9

## Conclusion and implications of the study

The experience of first-year SLP students engaging in teaching and learning through online platforms during the COVID-19 has been described. The majority of participants reflected that their online learning through the pandemic in 2021 was successful and that they did learn a lot during the year. Participants indicated that prerecorded lectures, weekly active engagement tasks and having supportive educators impacted their learning journey positively. These learning tasks are consistent with constructivist learning theory, as they promote students constructing their knowledge during the learning process (Khalaf & Zin, 2018). It is important for educators to consider how to integrate constuctivist learning theory prinicples when designing their courses to ensure the use of strategies that promote active learning and yet do not overwhelm students who are experiencing a great deal of adversity, stress and changes as a result of studying via online learning in a pandemic. This awareness will assist in transforming the pedagogies employed in teaching and learning so that a diverse group of students can be supported through the hybrid learning approach that the University of the Witwatersrand will be embarking on in 2022, as learning continues in a pandemic with all of its stress, changes, adversities and uncertainties.

It is also essential to consider the evolving support required from students across a year. The themes that emerged from this year pertained to 2021 and the specific participants; however, they provide an important insight – that students’ needs change. As a lecturer, one needs to consider these evolving needs to ensure students have the support that they require to be successful in their learning. It is also important to note that for each block, although there were challenges because of online learning during a pandemic, students reflected on support strategies, coping mechanisms and building resilience that supported them to experience successful learning and academic success during the specific block. Educators should foster a humanistic, caring, empathetic environment, as suggested by Cénat et al. (2020), one that can allow students to develop support strategies, coping mechanisms and ultimately resilience so that they can be successful learners navigating online learning in a pandemic. Developing their support strategies, coping mechanisms and resilience could support them in their adjustment to first-year tertiary education as well as for their future years of study. Whilst this study provided valuable information regarding first-year students’ experiences of online learning, it would be valuable to study these experiences with a different cohort of first-year students to look for any commonalities or differences, as well as studying the different learning needs of students across the years of study.

## Appendix 1: Likert scale to measure the first-year students’ experience

This Likert scale was developed based on the aims of the research to investigate the first-year students’ experience. The scale was sent to them via Google Forms.

**Instructions:**

Please reflect on the past year and specifically the SPPA1004A course when selecting the term on the scale that describes your perceived level of agreement with the statement. The terms will be the same for each item and will be organised as a scale under each statement. The terms are: strongly agree, agree, neutral, disagree and strongly disagree. Remember this will be anonymous and will not be linked to you or your results, so please be as honest as possible.

**Statements:**

1. I feel that online learning during my first year as an SLP student was successful.
2. Live lectures were beneficial in my learning journey.
3. Watching prerecorded lectures was beneficial in my learning journey.
4. Engaging weekly with structured tasks such as discussions, reflections and questions helped me learn better.
5. Not being able to meet my lecturers face to face hindered my learning journey.
6. Engaging in small group activities on Microsoft Teams breakout rooms did not support my learning journey.
7. Engaging in weekly activities was overwhelming.
8. I generally felt supported by my lecturers in SPPA1004A throughout the year.
9. I was able to manage my time so that I could complete all the work necessary.
10. I am confident that I learned a lot during my first year through the course SPPA1004A.

## Appendix 2: Timeline template for students to complete

**Instructions for students:**

Dear students,

Below is a template of a timeline. Please reflect over the past year and think of any incidents (academic or personal) that occurred in each block. These incidents should be things that occurred that had a significant positive or negative impact on you as a student. Please write in some detail as to what led up to that incident, and then write what was the consequence of that incident. Please also reflect on the reason that each of the incidents was important to you.
